# Dr. Harry W. Barber

**Published:** 1915-12

**Authors:** 


**Dr. Harry W. Barber,** University of Vermont 1906, honor man of his class, died at his home in Rochester, of angina pectoris, November 2, aged 35. He had already attained prominence as a surgeon, and was Assistant Surgeon and Anaesthetizer to St. Mary's Hospital, to which he had come as interne January 1, 1907. He was a brother of Dr. Leon Barber of Willard.

